# Recognizing Euglycemic Diabetic Ketoacidosis in the Era of SGLT2 Inhibitors: A Case Report

**DOI:** 10.7759/cureus.114492

**Published:** 2026-08-13

**Authors:** Jose C Serrano Reyes, Maria F Carrillo, Rolando L Jaen, Astrid J Batista

**Affiliations:** 1 General Practice, Emergency Department, Hospiten Paitilla, Panama City, PAN; 2 Internal Medicine, Emergency Department, Hospiten Paitilla, Panama, PAN; 3 Internal Medicine, Emergency Department, Hospiten Paitilla, Panama City, PAN

**Keywords:** diabetes mellitus type 2, diabetic ketoacidosis, diabetic ketosis, empaglifozin, euglycemic diabetic ketoacidosis, sodium-glucose cotransporter 2 (sglt2) inhibitors

## Abstract

Euglycemic diabetic ketoacidosis (euDKA) is an increasingly recognized complication associated with SGLT2 inhibitors. Because serum glucose levels may remain normal or only mildly elevated, diagnosis can be delayed, potentially increasing morbidity. We present the case of a 51-year-old male with long-standing poorly controlled type 2 diabetes mellitus who developed euDKA shortly after initiation of empagliflozin/metformin therapy. The patient presented with generalized weakness, gastrointestinal symptoms, reduced oral intake, polyuria, polydipsia, and weight loss. Initial laboratory evaluation revealed high anion gap metabolic acidosis with positive urinary ketones and a glucose level of 160 mg/dL. Infectious workup was negative. The patient required intensive care management with intravenous fluids, insulin infusion, electrolyte monitoring, and dextrose-containing fluids, with subsequent complete resolution of metabolic abnormalities. This case adds to the growing body of literature describing euDKA in patients receiving SGLT2 inhibitor therapy in the setting of reduced oral intake and poorly controlled diabetes mellitus.

## Introduction

Diabetic ketoacidosis (DKA) is a potentially life-threatening metabolic emergency classically characterized by hyperglycemia, metabolic acidosis, and ketosis [1,2]. DKA associated with SGLT2 inhibitor use has been reported to occur at an incidence of approximately 0.5 cases per 1,000 patient-years among patients with type 2 diabetes mellitus [2]. However, a subset of patients may develop DKA with normal or only mildly elevated serum glucose levels, a condition known as euglycemic DKA (euDKA). Although uncommon, euDKA has gained increasing clinical relevance with the widespread use of SGLT2 inhibitors [2,3].

SGLT2 inhibitors reduce plasma glucose levels through glycosuria while simultaneously promoting a metabolic state favoring ketogenesis through reductions in insulin secretion, increased glucagon activity, enhanced lipolysis, and increased free fatty acid oxidation. Several precipitating factors have been associated with euDKA, including prolonged fasting, dehydration, infection, surgery, reduced oral intake, vomiting, and insulin deficiency [1,4,5]. The absence of marked hyperglycemia often delays diagnosis because patients may initially appear less critically ill than those with classic DKA [2,3]. 

We present the case of a patient with poorly controlled type 2 diabetes mellitus who developed euDKA shortly after initiation of empagliflozin therapy in the setting of gastrointestinal symptoms and reduced oral intake.

## Case presentation

A 51-year-old male with a history of long-standing type 2 diabetes mellitus and hypertension presented to the emergency department with generalized weakness, dyspepsia, gastroesophageal reflux, abdominal bloating, and early satiety. His outpatient medications included metformin 850 mg twice daily for diabetes and perindopril 10 mg once daily plus amlodipine 10 mg once daily for hypertension. The patient also reported progressive polyuria, polydipsia, unintentional weight loss, and decreased oral intake over the preceding week. He denied fever or respiratory symptoms but reported two episodes of vomiting within the 24 hours prior to presentation. 

Ten days before admission, his outpatient diabetes regimen was changed from metformin monotherapy to empagliflozin combined with metformin because of persistently poor glycemic control. Outpatient laboratory studies obtained approximately three weeks before presentation demonstrated a fasting plasma glucose of 272 mg/dL and a hemoglobin A1c between 10.4% and 11%, consistent with markedly uncontrolled diabetes.

On arrival, the patient was hemodynamically stable but appeared clinically dehydrated with mild tachycardia. He was alert and oriented with a Glasgow Coma Scale score of 15. Cardiopulmonary examination was unremarkable. Abdominal examination revealed no tenderness or signs of peritoneal irritation. No peripheral edema was noted.

Initial laboratory evaluation demonstrated high anion gap metabolic acidosis with a venous pH of 7.16, serum bicarbonate of 9 mmol/L, serum glucose of 160 mg/dL, lactate of 1.4 mmol/L, creatinine of 1.35 mg/dL, blood urea nitrogen of 24 mg/dL, sodium of 135 mmol/L, potassium of 5.8 mmol/L, and chloride of 103 mmol/L. The calculated anion gap was 23. Urinalysis revealed positive urinary ketones without evidence of urinary tract infection. Complete blood count showed no leukocytosis, although hemoconcentration was present with a hematocrit of 47%.

**Figure 1:**
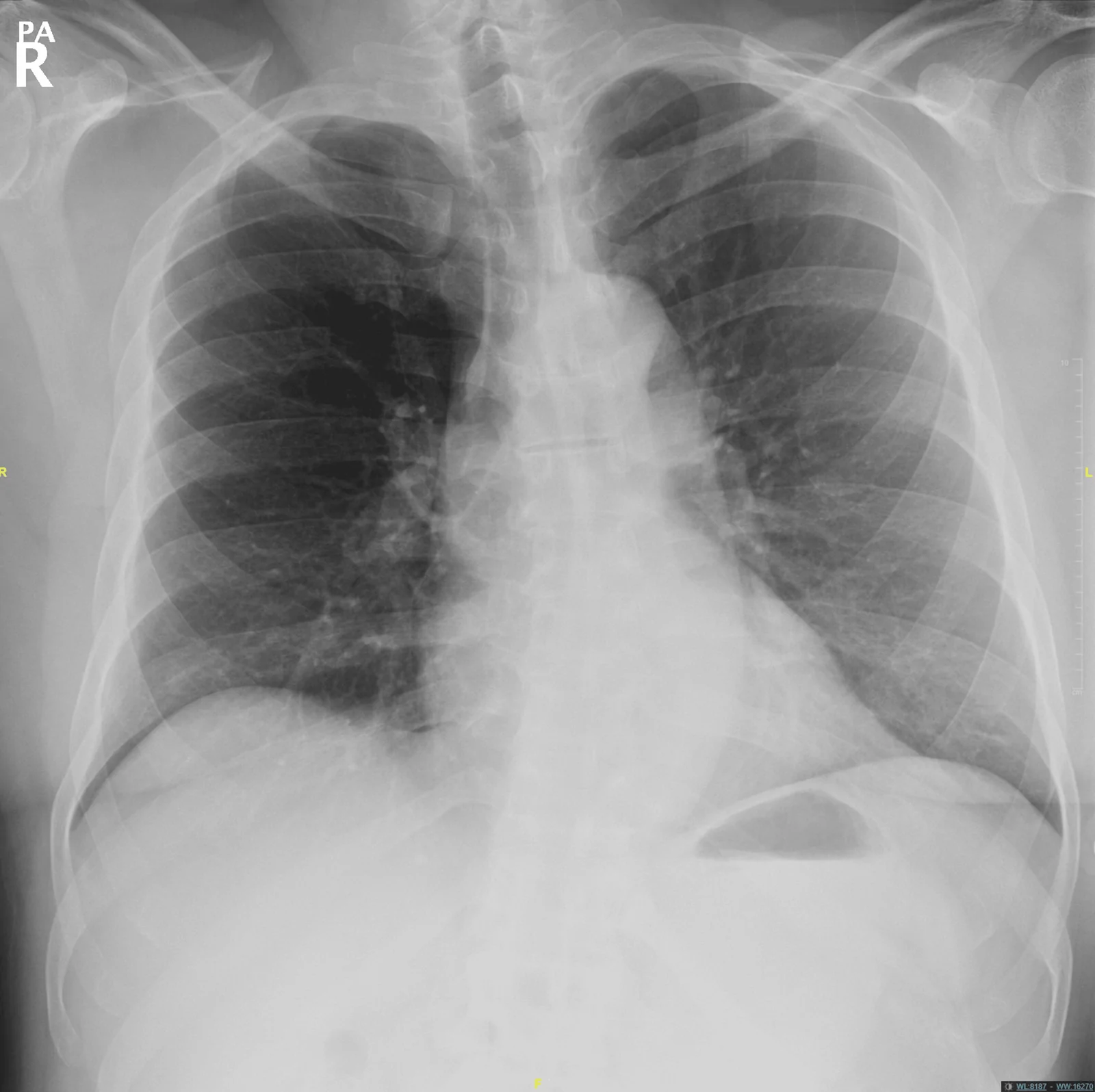
Chest radiograph obtained during the initial evaluation demonstrating no focal pulmonary infiltrates, pleural effusion, or other acute cardiopulmonary abnormalities.

An infectious workup was performed to evaluate potential precipitating factors. The chest radiograph showed no infiltrates or focal abnormalities (Figure 1), urinalysis was not suggestive of urinary tract infection, and procalcitonin was within the normal range. Blood and urine cultures subsequently remained negative, supporting the absence of an infectious precipitating factor.

The patient was diagnosed with euDKA in the setting of recent initiation of SGLT2 inhibitor therapy, reduced oral intake, and poorly controlled diabetes mellitus.

Intrahospital course

Hospital Day 1

The patient was admitted to the intensive care unit and treated with aggressive intravenous crystalloid hydration, dextrose-containing saline infusion, continuous intravenous regular insulin infusion, electrolyte monitoring, thromboprophylaxis with enoxaparin, and symptomatic gastrointestinal management with esomeprazole, metoclopramide, and granisetron.

Hospital Day 2 (Morning)

The patient remained hemodynamically stable with progressive improvement in metabolic acidosis. Follow-up laboratory evaluation demonstrated improvement in venous pH, serum bicarbonate, and lactate levels (Table 1). Oral intake was initiated and tolerated without recurrence of vomiting or abdominal pain.

**Table 1:** Serial laboratory results during hospitalization with corresponding reference ranges. Serial laboratory results demonstrating the evolution of metabolic parameters following initiation of treatment for euglycemic diabetic ketoacidosis. Reference ranges correspond to commonly accepted adult laboratory values and may vary slightly among institutions. *Hospital Day 4 glucose was measured after resolution of diabetic ketoacidosis and before hospital discharge.

Parameter	Reference Range	Hospital Day 1	Hospital Day 2 (AM)	Hospital Day 2 (PM)	Hospital Day 4
Glucose (mg/dL)	70–99 (fasting)	160	148	151	122*
Venous pH	7.35–7.45	7.16	7.29	7.30	7.37
Bicarbonate (mEq/L)	22–29	9	16	21	22
Lactate (mEq/L)	0.5–2.2	1.4	0.6	1.0	—
Sodium (mEq/L)	135–145	135	—	—	139
Potassium (mEq/L)	3.5–5.0	5.8	—	—	3.8
Chloride (mEq/L)	98–107	103	—	—	109
Creatinine (mg/dL)	0.6–1.3	1.35	—	—	0.60
Blood Urea Nitrogen (mg/dL)	7–20	24	—	—	—
Anion Gap (mEq/L)	8–12	23	—	—	8

Hospital Day 2 (Evening)

Continued metabolic improvement was observed (Table 1). Given his favorable clinical course, basal insulin therapy with insulin glargine was initiated in preparation for discontinuation of the intravenous insulin infusion.

Hospital Day 3

Following marked clinical improvement and laboratory findings from the previous day consistent with resolution of DKA (Table 1), the patient was transferred from the intensive care unit to the general medical ward. The intravenous insulin infusion was discontinued, and the patient was transitioned to subcutaneous insulin glargine. He remained clinically stable thereafter.

Hospital Day 4

Laboratory evaluation confirmed normalization of acid-base status, closure of the anion gap, and recovery of renal function (Table 1). Mild residual hyperchloremia was attributed to intravenous fluid resuscitation. The patient was discharged on insulin glargine 18 units daily, metformin 1000 mg twice daily, perindopril 10 mg daily, amlodipine 5 mg twice daily, and rosuvastatin 10 mg daily. Empagliflozin was discontinued. After receiving education regarding insulin administration and diabetes management, he was discharged home in stable condition.

## Discussion

euDKA is a recognized complication associated with SGLT2 inhibitor therapy. Unlike classic DKA, euDKA presents with normal or only mildly elevated serum glucose levels, typically below 250 mg/dL, which may contribute to diagnostic uncertainty [2,3]. 

DKA results from absolute or relative insulin deficiency, leading to increased lipolysis, hepatic ketogenesis, and high anion gap metabolic acidosis [1]. In SGLT2 inhibitor-associated euDKA, these metabolic abnormalities occur despite normal or only mildly elevated plasma glucose concentrations.

Under normal physiological conditions, insulin suppresses lipolysis and hepatic ketogenesis. Patients with diabetes who retain adequate pancreatic β-cell function usually maintain sufficient endogenous insulin secretion to prevent excessive ketone production. In contrast, individuals with reduced β-cell reserve, particularly those with long-standing type 2 diabetes who have progressive β-cell dysfunction and relative insulin deficiency, are more susceptible to ketoacidosis because they cannot mount an adequate compensatory insulin response during metabolic stress. The proposed mechanism of SGLT2 inhibitor-associated euDKA involves enhanced urinary glucose excretion, which lowers plasma glucose concentrations and consequently reduces glucose-stimulated endogenous insulin secretion. In patients with limited β-cell reserve, this further decreases circulating insulin levels, diminishing the inhibitory effect of insulin on pancreatic α-cells and contributing to increased glucagon secretion, thereby reducing the insulin-to-glucagon ratio. This hormonal imbalance promotes lipolysis, hepatic β-oxidation of free fatty acids, and excessive ketone body production. Additional precipitating factors, including prolonged fasting, reduced carbohydrate intake, dehydration, acute illness, surgery, or insulin dose reduction, further amplify ketogenesis. Because ongoing glucosuria limits hyperglycemia despite continued hepatic glucose production, patients may develop severe high anion gap metabolic acidosis with only mildly elevated or even normal plasma glucose concentrations, resulting in euDKA [2,4-6]. 

Several factors likely contributed to the development of euDKA in this patient. Recent initiation of empagliflozin occurred in the setting of markedly poor glycemic control (hemoglobin A1c 10.4%-11.0%), together with approximately one week of nausea, vomiting, dyspepsia, early satiety, reduced oral intake, dehydration, and weight loss. These factors likely acted synergistically to precipitate euDKA.

Alternative causes of high anion gap metabolic acidosis were evaluated and considered unlikely. Lactate levels remained within normal limits, infectious evaluation was unrevealing, and renal dysfunction was mild and rapidly reversible following treatment. Although serum beta-hydroxybutyrate measurements were unavailable, the combination of metabolic acidosis, ketonuria, and mild hyperglycemia remained consistent with previously described diagnostic features of euDKA [2,6-9]. 

The patient responded favorably to intravenous fluid resuscitation, continuous insulin infusion, electrolyte monitoring, and dextrose supplementation. Progressive normalization of acid-base status was observed during hospitalization, with closure of the anion gap from 23 to 8, improvement in serum bicarbonate from 9 to 22 mmol/L, normalization of pH from 7.16 to 7.37, and recovery of renal function, with creatinine decreasing from 1.35 mg/dL to 0.6 mg/dL at discharge. This favorable response is consistent with the standard management approach for DKA described in current guidelines [10-11]. 

This case describes the occurrence of euDKA following recent initiation of empagliflozin in a patient with poorly controlled type 2 diabetes mellitus, gastrointestinal symptoms, reduced oral intake, and dehydration. The diagnosis was established despite only mildly elevated serum glucose levels and was supported by the presence of high anion gap metabolic acidosis, positive urinary ketones, and complete resolution following standard treatment for DKA.

This case describes the occurrence of euDKA following recent initiation of empagliflozin in a patient with poorly controlled type 2 diabetes mellitus, gastrointestinal symptoms, reduced oral intake, and dehydration. The diagnosis was established despite only mildly elevated serum glucose levels and was supported by the presence of high anion gap metabolic acidosis, ketonuria, and complete resolution following standard treatment for DKA. Although this case involved empagliflozin, euDKA has been reported with other SGLT2 inhibitors, including canagliflozin, dapagliflozin, ertugliflozin, and sotagliflozin, and is considered a class effect. In addition to this potentially life-threatening complication, clinicians should remain aware of other recognized adverse effects of SGLT2 inhibitors, including genital mycotic infections, urinary tract infections, volume depletion, and the rare occurrence of Fournier's gangrene [12]. 

## Conclusions

EuDKA is a potentially life-threatening complication of SGLT2 inhibitor therapy. The absence of severe hyperglycemia may delay diagnosis and treatment. Clinicians should maintain a high index of suspicion, particularly in patients with poorly controlled diabetes, reduced oral intake, dehydration, or gastrointestinal symptoms following initiation of SGLT2 inhibitors. Early recognition and prompt management are critical to achieving favorable outcomes.
